# Spillover Effects of Food Safety Incidents: Role of Consumers’ Heterogeneous Safety Preferences

**DOI:** 10.3390/foods14173085

**Published:** 2025-09-02

**Authors:** Fang Ren, Jin Fan

**Affiliations:** 1 College of Economics and Management, Nanjing Forestry University, Nanjing 210037, China; anna_njfu@163.com; 2 School of Finance and Accounting, Henan University of Animal Husbandry and Economy, Zhengzhou 450044, China

**Keywords:** food safety incidents, security preference, risk perception, general equilibrium model, food safety level, market share, market risk

## Abstract

This study considers consumers’ risk perceptions and safety preferences as external shock factors in food safety incidents. These factors are incorporated into a general equilibrium model defined by the food safety hierarchy, and the computational experiment method is employed to examine the direction of spillover effects. According to the findings, the spillover direction and intensity of food safety incidents are jointly influenced by the characteristics of consumers, food and the market. When an incident raises consumers’ safety concerns, a negative effect occurs throughout all food sectors. When an incident has a specific impact on consumers’ risk perception, the direction of the spillover is contingent upon the safety level of the product in question. In the event that the food involved in an incident is extremely secure, it may have a detrimental effect on unrelated food goods; conversely, it may have a beneficial effect on unrelated food goods. The incident’s impact has increased in proportion to the market share of the affected food. When the market share remains constant, the impact intensity increases as the degree of food safety improves. Higher market-wide risk levels are associated with more pronounced and quicker effects. This study improves understanding of spillover patterns in food safety situations, which aids in the formulation of focused policy responses and initiatives.

## 1. Introduction

Major food safety incidents not only affect the directly implicated products but also exert a “spillover effect” on unrelated food categories [1]. For instance, the food poisoning incident involving Chinese imported dumplings in Japan reduced the proportion of consumers intending to purchase Chinese food from 92.5% to 27.6% [2]. Similarly, in China, dairy consumption declined sharply following the melamine contamination incident [3,4]. Such events have led to substantial economic losses [5,6,7]. Research shows that exposure to various informational stimuli affects consumers’ perceptions of food safety and preferences, leading to changes in consumption patterns [8,9,10]. The willingness to purchase has fallen dramatically [1,11,12,13]. This phenomenon has two effects: it impairs the quality of directly involved food products and has a “spillover effect” on non-related food goods [14]. Food safety incidents have a substantial impact on food market volatility frequency, restricting government macro-control power.

The spillover impacts of food safety events are diverse [14]. Research perspectives vary: some scholars identify positive spillover effects [2,15,16], others observe negative spillover effects [1,17]. However, obtaining sufficient profits is the primary goal for the survival of enterprises. In existing studies, there are few analyses on the direction of the impact of events on enterprise profits. Is it necessarily beneficial to the enterprise that the price or sales volume of non-involved food increases after the incident? Some studies indicate indeterminate spillover directions [18]; however, the differences in the levels of food safety involved were overlooked. Therefore, would the disparities in the safety levels of the food in question also result in varying intensities and orientations of the impact? Research shows that consumers’ purchasing behavior after food safety incidents connects with their risk perception and preference [19,20,21,22]. Thus, investigating consumers’ risk perceptions and safety preferences improves explanatory power for spillover direction variability. This poses several key questions. How do people’s risk perceptions and safety preferences affect the direction and magnitude of food safety spillovers? Given population variation in risk perception and safety preferences, what patterns emerge in food safety spillover directions? Do events provide consistent spillovers in pricing, volume, and profit metrics? Addressing these concerns is helpful in revealing the fluctuation patterns of non-involved food prices, sales volumes, and profits under the impact of uncertain events, as well as in clarifying the differences in spillover directions when the safety levels of the involved food differ. This provides strategic guidance for enterprise risk management and informs government policy development for diversified food market regulation.

## 2. Literature Review

Existing research from various countries on food safety incident spillover directions focuses on competitive interactions between affected food products. Several studies have found that detrimental negative effects on individual factors such as sales volume and price of competing enterprises within the same industry [1,12,23,24,25,26]. For example, in the United States, the recall of *E. coli* in beef significantly reduced concurrent demand for recalled ground beef in most (but not all) regions of the United States [27]; in Japan, following the poisoning event involving Chinese imported dumplings in Japan, the number of consumers planning to buy Chinese food dropped by over two-thirds [2]; and in China, following the melamine incident, Chinese consumers’ consumption of dairy products fell sharply [3,4]. Conversely, some studies indicate positive spillover effects for competing brands and enterprises within the same industry [17]. Examples include increased consumer preference for pasteurized milk and yogurt following the melamine incident [28], as well as a greater preference for foreign brand milk powder [29], and increased demand for imported pork following domestic pork safety incidents [16]. 

Food safety issues often have a beneficial effect on the sales volume, prices and even stock prices of non-involved food sector enterprises [30,31]. For example, mutton prices rose in response to pig epidemic outbreaks in China [32]; one week after the mad cow disease incident in the United States, the share prices of beef competitors in the capital market rose significantly [33]; and in 2017, the Spanish tuna fraud incident caused an increase in the price of cod [17]. To summarize, the outbreak of food safety incidents has prompted consumers to be willing to pay a premium for truly safe food [29,34,35] and to pay the differential premium level based on the food’s safety attributes [36]. However, other studies indicate that the impacts on replacement production enterprises are questionable [37]. Li, Meiqi, and colleagues [18] investigated geographical variations in the African swine disease epidemic’s impact on alternative meat pricing (beef, mutton, and chicken).

There is a direct correlation between the severity of spillover effects and consumers’ perceived risk and safety preferences [2]. In response, consumers may switch to alternative food products from other industries, resulting in adverse impacts on the affected industry while benefiting unaffected sectors [31,38,39,40]. The product’s position within its category also shapes the nature of spillover. Crisis events associated with severe harm, widespread occurrence, or connections to prominent enterprises exert a stronger influence on the industry as a whole [18,41].

As shown in the previous analysis, the existing research has investigated the heterogeneity of the spillover direction and severity of food safety incidents, as well as the contributing factors. However, four major holes remain. First, although existing studies have found heterogeneity in the direction of event spillover, they have not explained the reasons. The present research does not provide a full study of how citizens’ risk perception and safety choices influence results. While previous research has addressed the impact of risk perception and safety preferences on consumers’ risk behaviors, the underlying mechanisms have not been adequately investigated. Second, there is limited research on the variability of event impacts on food products at various safety levels, as well as the factors that influence their occurrence. Current research focuses mostly on brand and category impact heterogeneity, with little attention paid to comprehensive security analysis. Third, there is an insufficient multi-index, thorough study of event effect results. Most studies focus on single parameters, such as pricing and sales volume, while ignoring profit analysis. However, profit maximization remains central to business operations. During unforeseen events, both company operations and government regulatory laws necessitate a thorough understanding of numerous components’ influence patterns. As a result, a post-impact study of various components’ directional shifts is critical. Fourth, the existing research primarily employs event study methods, using econometric models such as multiple regression, the QUAIDS model, and the structural equation model to investigate specific events or food safety incidents. However, consumers’ safety preferences and risk perception levels vary across geography and time, leading to significant differences in purchasing decisions. Even large-sample empirical approaches have limitations. Post-incident food demand and enterprise output equilibrium constitute a complex system. The general equilibrium model offers advantages in incorporating numerous agents and factor changes, while computational experimentation enables the simulation of complex multi-agent interactions [42]. As a result, this research presents a multi-agent general equilibrium model for simulating and analyzing abrupt food safety event spillover direction and intensity through computer experimentation.

**Figure 1:**
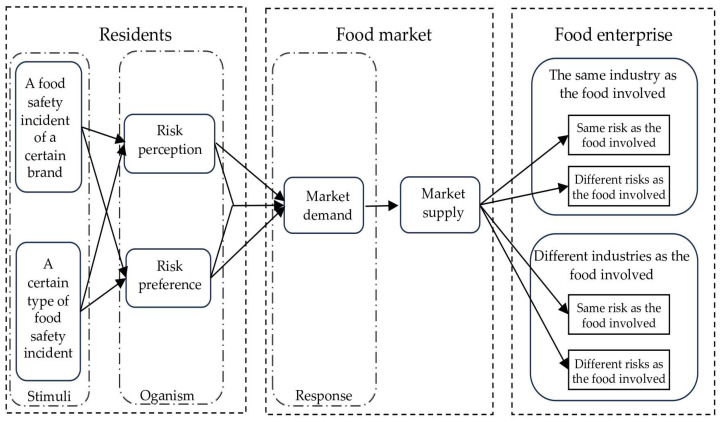
The mechanism of residents’ risk perception and risk preference on the spillover effect of food safety incidents.

According to the stimulus-organism-response (SOR) model, when a food safety incident occurs, consumers’ risk perceptions and safety preferences are altered by the stimulus of the incident. These changes are reflected in their purchasing behavior, which manifests as adjustments in the composition of food demand across different safety levels. Based on general equilibrium theory, the equilibrium price, sales volume, and profit of food products at varying safety levels are determined by the interaction between consumers’ demand and enterprises’ production decisions. To investigate the spillover direction of food safety incidents under shifts in consumers’ risk perceptions and safety preferences, this study integrates the SOR model with the general equilibrium framework to construct a model assessing the impact of food safety incidents on the equilibrium outcomes of the food market. The model takes into account resident safety preferences as well as corporate product safety heterogeneity. It simulates the dynamic evolution of consumers’ consumption patterns across various safety levels, as well as the related enterprise production, in response to an unforeseen food safety incident. The study investigates the “spillover” direction of food safety incidents on different non-involved food products, as well as the factors that influence spillover direction and heterogeneity strength. In addition, the research makes policy recommendations for government shock reaction, “food basket” stabilization, and enterprise production decisions. Figure 1 shows the research logic for this work.

This paper’s marginal contribution includes a few crucial aspects: First, it examines the process for producing food safety spillover heterogeneity by combining consumers’ safety preference heterogeneity with food safety heterogeneity. The study found that different impact factors on consumers result in different spillover effect orientations. Second, it investigates spillover impact heterogeneity from a food safety hierarchy standpoint, indicating that different safety levels of included food products result in diverse incident spillover paths. Third, it conducts a thorough analysis of food safety incident impacts across various dimensions, including price, demand, and profit, finding diverse spillover directions for different indicators within the same event. Fourth, the intensity and duration of event shocks were also determined by the risk level of the market, as evidenced by the discussion of the consequences of event shocks in various risk markets. These findings considerably supplement previous research.

## 3. Basic Model Construction

The model developed in this study extends the framework proposed by Daughety and Reinganum [43] by introducing consumers’ risk perceptions and safety preferences as shock factors. It is used to analyze the direction and intensity of their effects on the prices, sales volumes, and profits of products from non-involved enterprises following the outbreak of food safety incidents. Assuming constant marginal costs, the market consists of *n* enterprises, each producing one type of food with different safety levels or different production costs. A representative consumer selects a commodity bundle consisting of *n* types of food according to their food safety preferences. For analytical clarity, the numerical simulation presented in this paper simplifies consumer choices into two scenarios. In Scenario One, foods differ in safety level; the consumer’s bundle consists of *n* products with varying safety levels and costs. In Scenario Two, foods share the same safety level, and the consumer’s bundle consists of *n* products with identical safety levels but different costs.

### 3.1. Maximizing Consumer Utility

Representative consumers choose *n* (*n* ≥ 2) types of food with differing safety levels based on their food safety risk preferences. According to the SOR model, food safety incidents act as external stimuli that influence consumers’ risk perceptions and safety preferences, thereby shaping their purchasing behavior toward safer food products. Based on the models of Daughety and Reinganum [43] and Zhu et al. [44], which characterize utility in terms of food consumption needs and food safety behaviors, the maximization of food consumption utility for typical consumers can be defined as follows:

(1) $$U\left(x_{1},x_{2,}\cdots x_{n}\right)=\sum_{i=1}^{n}\left(\alpha -\gamma F_{i}\right)x_{i}-\frac{1}{2}\beta \sum_{i=1}^{n}x_{i}^{2}-\frac{1}{2}\vartheta \sum_{i=1}^{n}\sum_{j\neq i}^{n}x_{i}x_{j}\;\;s.t.\sum_{i=1}^{n}p_{i}x_{i}<I$$

Among them, Ω represents the set of available foods, $x_{i}$ is defined as the consumption of food item *i* by the population, $p_{i}$ is defined as the price of food item *i*, $F_{i}$ is the safety risk level of food item *i*, and I is the income of consumers. The parameter γ represents the food safety behavior of consumers. The smaller the γ, the more consumers prefer to purchase food with a lower risk level, that is, the stronger the degree of consumers’ safe food consumption behavior. β(>0) is the consumer’s preference for diversity, and ϑ∈(0,β) is the competitive strength of two foods with different safety levels. Consumers choose food combinations of different safety levels under the budget constraints $\sum_{i=1}^{n}p_{i}q_{i}<I$ to maximize their utility. Through calculation and organization, the demand function of consumers for the *i*-th type of food can be obtained:

(2) $$x_{i}=\frac{a-c(X-P)-b\gamma F_{i}-(\lambda b+c)p_{i}}{1-c}=\frac{d-b\gamma F_{i}-(\lambda b+c)p_{i}}{1-c}$$

Among them, $a=\frac{\alpha }{\beta +(n-1)\vartheta }$, $b=\frac{\beta +(n-2)\vartheta }{\left(\beta -\vartheta \right)[\beta +\left(n-1\right)\vartheta ]}$, $c=\frac{\vartheta }{\left(\beta -\vartheta \right)[\beta +\left(n-1\right)\vartheta ]}$, d=a−c(X−P), and $X=\sum_{i=1}^{n}x_{i}$, $P=\sum_{i=1}^{n}p_{i}$. The equation describes the facts about food consumption among consumers: food with low risk ($F_{i}$), low price ($p_{i}$), and high variance (smaller ϑ) is more popular among consumers and has greater market demand.

### 3.2. Maximizing Enterprise Profits

Suppose each enterprise produces different food *i*, and its output is $q_{i}$(i∈Ω). Safe production can reduce the losses of enterprises in food safety incidents [45]. Increasing investment in safety costs is an important guarantee for improving the level of food safety [46,47]. This article assumes that the cost of food consists of two parts: production cost and safety cost. To simplify the discussion process, drawing on the practices of Napel and Oldehaver [48], Eckel et al. [49], and Zhu et al. [44], it is considered that the link between the investment cost of food safety production and the level of food safety risk corresponds to $F_{i}^{-1}=\sqrt{\varphi_{i}C_{fi}}$, where φ>0 represents the food safety conversion rate of the enterprise. Furthermore, drawing on the practices of Bureau et al. [50], let the production cost of the enterprise be $\frac{1}{2}c_{i}q_{i}^{2}$. This leads us to the conclusion that the investment cost of food safety for enterprises is

(3) $$C_{fi}=\frac{F_{i}^{-2}}{\varphi_{i}}$$

The enterprise’s total profit is

(4) $$\pi_{i}=p_{i}q_{i}-\frac{1}{2}c_{i}q_{i}^{2}-\frac{F_{i}^{-2}}{\varphi_{i}}$$

At equilibrium, resident demand equals enterprise output. Substituting Equation (2) into Equation (4) yields the following:

(5) $$\pi_{i}=\frac{d-b\gamma F_{i}}{1-c}p_{i}-\frac{\left(\lambda b+c\right)}{1-c}p_{i}^{2}-\frac{1}{2}c_{i}{\left(\frac{d-b\gamma F_{i}-(\lambda b+c)p_{i}}{1-c}\right)}^{2}-\frac{F_{i}^{-2}}{\varphi_{i}}$$

### 3.3. Market Equilibrium

The market equilibrium is achieved when food prices and sales volumes that maximize consumer utility converge with profit-maximizing enterprise demand.

The partial derivative of the profit function with respect to $p_{i}$ yields the equilibrium price:

(6) $$p_{i}^{*}=\frac{\left[d-b\gamma F_{i}\right]\left[1+\left(\lambda b+c\right)c_{i}\right]}{[2+c_{i}\left(\lambda b+c\right)]\left(\lambda b+c\right)}$$

By substituting the equilibrium price into Equation (2) of the demand quantity function, the equilibrium output (demand quantity) can be derived.

(7) $$q_{i}^{*}=\frac{d-b\gamma F_{i}}{(1-c)\left[2+\left(\lambda b+c\right)c_{i}\right]}$$

The prerequisite for a food to have a positive price is $a+c\left(P-X\right)-b\gamma F_{i}>0$, and the prerequisite for having a positive sales volume is 1−c>0. This reflects the competitive fact of the food market:

Equations (6) and (7) are substituted into Equation (5), yielding the equilibrium profit function:

(8) $$\pi_{i}^{*}=\frac{{\left[d-b\gamma F_{i}\right]}^{2}\{2\left(1-c\right)+\left(1-2c\right)\left(\lambda b+c\right)c_{i}\}}{2\left(\lambda b+c\right){\left(1-c\right)}^{2}{\left[2+\left(\lambda b+c\right)c_{i}\right]}^{2}}-\frac{F_{i}^{-2}}{\varphi_{i}}$$

### 3.4. Analysis of the Impact of Sudden Food Safety Incidents on the Food Market Equilibrium

Sudden food safety accidents are distinguished by their unpredictability and quick escalation. Once such occurrences develop, businesses confront considerable problems in quickly altering their product safety levels or manufacturing procedures. The equilibrium model predicts a rapid increase in consumers’ food safety preferences (γ). Additionally, due to the elimination of foods failing to meet safety requirements, the number of market enterprises decreases (∆n < 0). Assuming that total resident food demand X remains constant, given its limited availability in reality, the remaining businesses must absorb all resident demand, upsetting the original equilibrium. As a result, prices, demand volumes, and profitability for various food categories fluctuate.

Initially, the total market price P undergoes short-term fluctuations. Given (1), it follows that

(9) $$P=\sum_{i=1}^{n}p_{i}=na-\left[\beta +\left(n-1\right)\vartheta \right]X-\gamma F$$

Following the food safety incident outbreak, the change in total food price P is

(10) $$∆P=∆n\left(\alpha -\vartheta X\right)-[(1+\eta_{\gamma })(1+\eta_{F})-1]\gamma F$$

Among them, $\eta_{\gamma }=\frac{∆\gamma }{\gamma }$, $\eta_{F}=\frac{∆F}{F}$. The shift in *P* following a shock is influenced by both consumers’ safety preferences (*γ*) and overall market risk (*F*). That is, the various risk structures in the food market are one of the causes contributing to the disparities in impact results.

From Equation (6), it is known that the variation of the equilibrium point price $p_{i}^{*}$ depends on $M_{1}=a+c\left(P-X\right)-b\gamma F_{i}$, and only when $M_{1}>0$ can there be a positive price. By (7), equilibrium sales $q_{i}^{*}$ depend on the change of $M_{2}=\frac{a+c\left(P-X\right)-b\gamma F_{i}}{1-c}$, and (1−c)>0 only have a positive sale. By (8), equilibrium profit $\pi_{i}^{*}$ changes depend on ${M_{3}=(M_{2})}^{2}\{2\left(1-c\right)+\left(1-2c\right)\left(b+c\right)c_{i}\}$ when $2\left(1-c\right)+\left(1-2c\right)\left(b+c\right)c_{i}>0$ has positive profits. Obviously, after the shock, the shifting directions of individual food prices, sales volumes, and profits are quite complex and influenced by a variety of factors, not just one.

## 4. Impact Analysis Based on the Heterogeneity of Consumers’ Food Preferences

The above analysis shows that food safety incidents affect both identical items with varied safety levels [16] and diverse food kinds with comparable safety standards. However, it is unclear whether the eventual outcome will appear as a positive effect or a negative effect. This section examines and discusses the impact on food groups with equal and varying safety standards.

**Figure 2:**
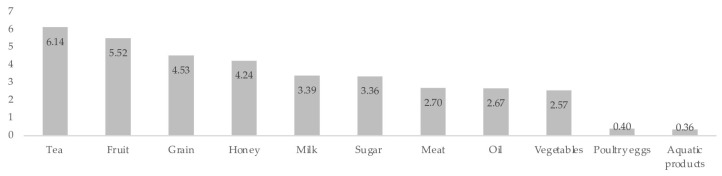
The green proportion of various types of food production in 2022.

Furthermore, the market risk structure differs dramatically among China’s food types (see Figure 2). In 2022, tea had the highest green rate (6.14% of total output), while aquatic items had the lowest (0.36%). Given China’s huge geographical spread, regional market risk structures differ significantly. The model derivation results show that the food market’s risk structure determines the direction of impact. As a result, this part calculates and analyses equilibrium value changes at each safety level in response to food safety incidents in both high-risk and low-risk markets, investigating the differences in post-impact outcomes between these two risk structures.

According to the theoretical model, consumers demonstrate a significant preference for diversity. Table 1 shows the market’s set basic parameter values. The experimental variables and parameters are established based on China’s current food quality certification system. The primary food quality certifications in China comprise pollution-free agricultural product certification, green food certification, and organic food certification, alongside a mandatory food production license system. Based on these certification standards, market food products can be categorized into five levels of safety, from highest to lowest: organic food, green food, pollution-free food, qualified food (with only a food production license), and substandard food (without a food production license). This study examines these five food safety categories, conducting simulations and analyses to evaluate the differential impacts of food safety incidents. Consumer food consumption exhibits natural limitations, remaining finite even with increased income. Consumption patterns vary significantly across food categories such as grains, edible oils, and vegetables. According to the “China Statistical Yearbook,” the average per capita food consumption for Chinese residents in 2024 is 39.78. This study adopts a total consumer food demand (X) of 40. Additional parameters are derived from model assumptions and conditions necessary for valid results. For instance, Equation (7) requires $a+c\left(P-X\right)-b\gamma F_{i}>0$ for positive pricing and 1−c>0 for positive sales volume. These parameters were refined through iterative testing to maximize alignment with real-world conditions.

The present food safety levels in China are generally classified according to certification marks, which range from lowest to highest: unqualified, qualified, pollution-free, green, and organic. Foods within the same grade may have varied safety standards due to variances in origin, processing, and brand identification, but these variations remain within the grade limitations. This article examines the impact of food safety at five risk levels: high (80 and 60), medium (30), and low (10 and 0.1). Higher safety standards imply higher expenses for monitoring, certification, and related activities. For computational simplicity, food with a risk rating of 60 is assigned a starting cost of one, with costs increasing proportionally with safety level improvements. Table 2 shows the relevant parameter settings. The initial food safety conversion rate for businesses is consistently set at 0.8. This paper utilizes the computational experiment method to determine and evaluate the influence of changes in a variety of factors on the equilibrium value. By substituting the parameter values into Equations (6)–(8), the equilibrium values corresponding to different variable values can be calculated and obtained.

### 4.1. Analysis of the Impact of a Group of Foods at Different Safety Levels

**Figure 3:**
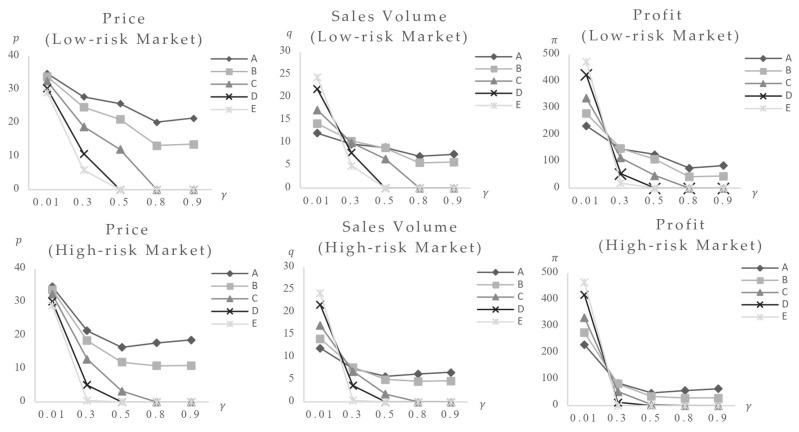
The changes in the equilibrium values of food at various safety levels as consumers’ safety.

1. Only the impact of consumers’ food safety preferences variation. The consumers’ food safety preferences γ are set at 0.01, 0.3, 0.5, 0.8, and 0.9. The model calculates equilibrium values for foods with varied safety levels (parameters listed in Table 2) following the effect in both low-risk and high-risk market structures. Figure 3 shows the results (note: Variable γ represents consumers’ safety preferences; Variable p represents market equilibrium price; Variable q represents market equilibrium sales volume; Variable π represents market equilibrium profit; *t* represents time. Descriptions of variable units for all following figures are given in Appendix B).

The graph shows that increasing consumers’ desire for food safety causes quick drops in equilibrium prices, sales volumes, and profits across all food categories, indicating a large negative effect. Foods with risk values of 80 and 60 exit the market at γ=0.5, while those with risk values of 30 exit at γ=0.8. Foods with risk values of 0.5 and 10 stabilize after γ reaches 0.8. This indicates the market’s self-regulatory capacity: eliminating high-risk foods and achieving a new equilibrium. However, markets with different risk structures respond differently to impact: (1) In high-risk markets, food with a risk value of 80 exits at γ = 0.3, whereas in low-risk markets, exit occurs at γ=0.5, indicating earlier elimination of higher-risk foods in high-risk markets; (2) foods with risk values of 0.5 and 10 in high-risk markets reach their lowest point and stabilize at γ = 0.5, while in low-risk markets, stabilization occurs at γ = 0.8, indicating faster shock processing in high-risk markets; (3) the high-risk market curves have greater slopes. A comparison of equilibrium values reveals that post-impact lowest prices for foods with risk values of 0.5 and 10 in high-risk markets are 16.40 and 11.94, respectively, which are lower than 20.22 and 13.22 in low-risk markets, showing more extreme shock-induced fluctuations in high-risk markets.

Additional post-impact research of market performance with category expanded fourfold (*n* = 1600) demonstrates that in markets with equal risk structures, category competition intensity impacts the size of food equilibrium values but has no effect on market trends. The market exit timing for diverse foods remains constant, as does the equilibrium value trend (numerical data and graphs are excluded).

These findings largely support Yi et al.’s [51] conclusion that isolated food safety incidents cause price increases, particularly in high-risk products. The distinction rests in finding that such accidents can also cause price increases in low-risk products. In contrast to high-risk products, the drop in price, sales volume, and profit is relatively limited, stabilizing as consumers’ safety preferences normalize.

According to this study’s findings, when an incident is not specific to a particular brand or food type and does not change consumers’ risk perception levels while increasing safety preferences, it has a negative effect on market equilibrium prices, sales volumes, and profits across food categories. In the end, the market removes high-risk items on its own. In markets with higher total hazards, high-risk foods face more severe consequences and are eliminated sooner. In contrast, low-risk market systems may allow high-risk foods to remain as chronic market issues. In the current body of research, no distinct study has been identified that examines the influence of changes in consumer safety preferences on the direction of spillover from food safety incidents. Consequently, the aforementioned conclusion serves as a valuable addition to the current body of inquiry.

2. Only the impact of food safety variation. Setting the consumers’ safety preference γ at 0.3, the model calculates the changes in food equilibrium values at each level as a result of one or more food safety incidents at various levels.

**Figure 4:**
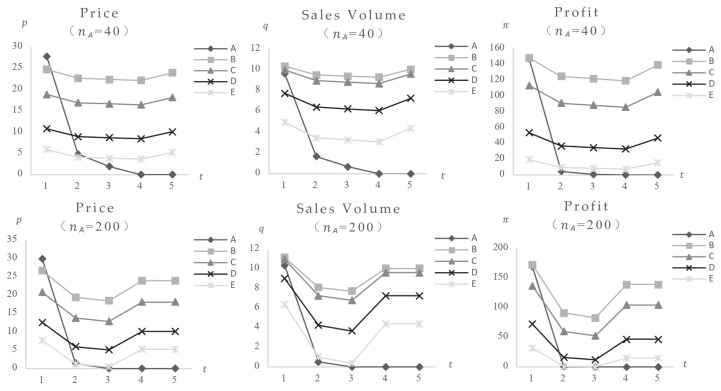
Safety incidents occur in food A ($F_{A}$ = 0.1), and the equilibrium values of foods at different safety levels change (γ=0.3).

**Figure 5:**
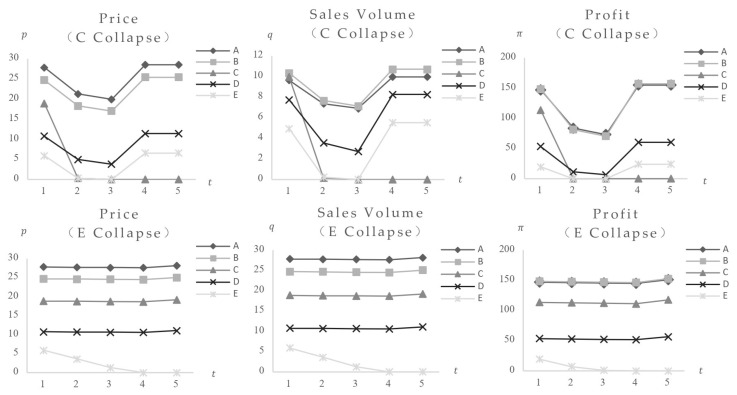
When safety incidents occur in Food C ($F_{C}$ = 30) or Food E ($F_{E}$ = 80), the changes in the equilibrium values of food at different safety levels (γ=0.3).

First, the research looks at the results of a food safety incident. Figure 4 shows that when the consumer safety preference is 30, the impact of a safety incident using Food A, which poses the lowest risk ($F_{A}$ = 0.1), as well as the variances induced by different market locations (categories). The data show that after Food A’s safety incident, its equilibrium value dropped quickly, while other foods initially reduced, then increased, eventually reaching a new stable state below pre-incident values, suggesting a definite negative effect. The comparison of impact charts for Category A with 40 and 200 categories shows that foods with higher market positions (category quantity) have greater market impact and expedite the impact process. Figure 5 shows the market impact of incidents involving medium-risk Food C ($F_{C}$ = 30) and high-risk Food E ($F_{E}$ = 80). The results show that following safety events in medium and high-level foods, the equilibrium values of remaining foods, despite an initial decrease followed by a rise, stabilize at levels higher than pre-incident levels, indicating a positive effect. Higher market positions have always been associated with greater market impact from food scandals.

In addition, calculations were made to determine the simultaneous collapse of two food varieties on the marketplace. The general trend was consistent with the previously stated impact results. Specific values are withheld.

These findings are consistent with those from empirical studies, food safety incidents boost the sales of safer food products. For example, after China’s “lean meat powder” and “melamine” episodes, consumers boosted their consumption of safer organic pig, foreign pork, and dairy products [16,28]. However, previous research has not addressed the diversity of spillover directions following accidents involving foods of varying safety levels. The findings of this work add significant value to previous studies.

The findings support multiple conclusions: When a food safety incident affects just consumers’ risk perception, such as when a specific brand or food type is involved, accidents involving high-safety goods have detrimental negative effects on foods at lower safety levels. Incidents involving high-risk foods, on the other hand, have a favorable positive effect on items with lower safety ratings. Furthermore, larger market placements (category quantities) of affected foods (both individual and combined) are associated with greater market impact and faster impact progression.

3. Shock with both consumers’ food safety preferences and food safety varying. The analysis considers consumers’ food safety preferences γ at 0.01, 0.3, 0.5, 0.8, and 0.9. The model calculates changes in equilibrium values at each food safety level as a result of one or more food safety incidents at various levels.

**Figure 6:**
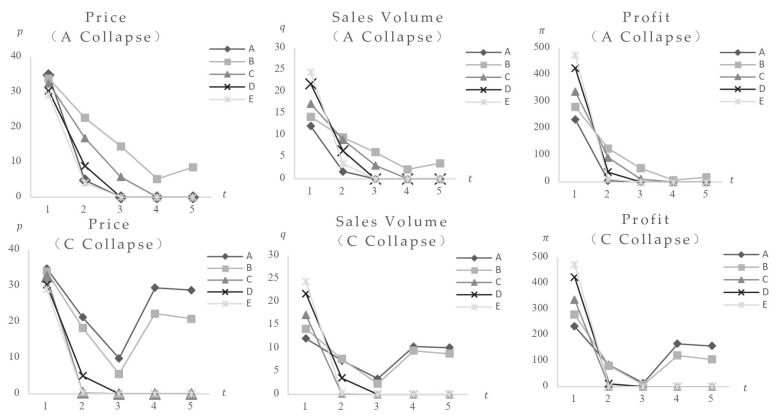
Both γ and *F* change, safety incidents occur in Food A ($F_{A}$ = 0.1) or C ($F_{C}$ = 30), the equilibrium values of foods at different safety levels change.

Similarly, the research begins by looking at individual food incidents and how they are impacted by changes in resident safety preferences. Figure 6 shows the effects of safety incidents involving low-risk Food A ($F_{A}$ = 0.1) and medium-risk Food C ($F_{C}$ = 30). The results indicate that after low-risk Food A failed, consumers’ safety preferences increased, resulting in lower volumes and prices for higher-risk Foods C, D, and E until they were eliminated. Lower-risk Food B experienced an initial decrease, followed by a rebound, before stabilizing below pre-incident levels. Following the failure of medium-risk Food C, rising safety preferences led to lower volumes and pricing for higher-risk Foods D and E, which were eventually eliminated. Reduced-risk Foods A and B had an initial decline, a subsequent increase, and a final decrease before reaching new stable states at reduced risk levels. A comparison of Foods A and C shock impacts suggests that, despite distinct impact procedures, final outcomes consistently exhibit new equilibrium values below pre-shock levels, counterweighting the negative effect. Other foods’ effect results follow similar patterns, with higher market positions corresponding to larger market impact; hence, the detailed results are ignored.

These data show that when food safety incidents alter both consumers’ risk perceptions and safety preferences, they have a considerable impact on foods at all market safety levels. High-safety food events cause extended market stagnation and difficult recovery. However, after high-risk food accidents, high-safety food prices, sales volumes, and profitability show a modest recovery potential. This conclusion is essentially in accordance with the previous one, which was reached when only consumer safety preferences were altered. However, the distinction is in the extent and rapidity of recovery following the shock.

### 4.2. Analysis of the Impact of a Group of the Same Security Level Foods

According to research, food safety incidents affect not just goods with variable degrees of safety, but also different brands and varieties of foods within the same safety category. For example, the 2017 Spanish tuna incident led to an increase in the price of pollock [17]. Given the different conclusions in the previous literature regarding the direction of influence, this section examines the effects on a set of identical F foods to evaluate the spillover effects of abrupt food safety incidents on other foods in the same safety category.

**Figure 7:**
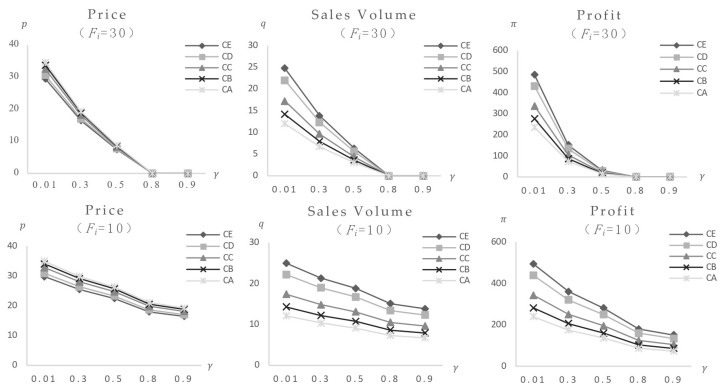
The equilibrium value of food at the same safety level changes only when consumers’ safety preferences increase.

1. Only the impact of consumers’ food safety preferences variation. The consumers’ food safety preferences γ are set at 0.01, 0.3, 0.5, 0.8, and 0.9. The model computes equilibrium values for a set of foods with identical safety levels ($F_{i}$ = 30) (parameter values reported in Table 3) after being exposed to both low-risk and high-risk market configurations. Figure 7 shows the results. The findings show that when consumers’ safety preferences rise, the equilibrium values of items with the same safety level initially fall and then stabilize post-shock, without rebounding, suggesting a negative effect, with high-risk foods suffering the most serious implications. Calculations of equilibrium values across high-risk markets and different categories yield consistent conclusions: when the event triggers only γ changes, the impact manifests as a negative effect, with greater impact correlating to higher food and market risk levels. Specific data and graphs are omitted for brevity.

According to the analysis, if a food safety incident just influences consumers’ safety preferences, it has a negative effect on all similar meals in that safety category.

In line with the findings in Figure 3, increased resident preference for the safety of a certain food type leads to decreased consumption of both same-safety-level and higher-safety-level food of the same type, with consumers turning to alternative food types.

2. Only the impact of changes in food safety. The model calculates changes in the equilibrium values of other foods as a result of safety incidents impacting one or more foods (with varied costs or types) within the same safety level group (parameter values reported in Table 3). The analysis assumes consumers’ food safety preference γ = 0.3, uniform food safety risk of 30, and variations in cost, price, and market position (quantity of categories) across the food group.

**Figure 8:**
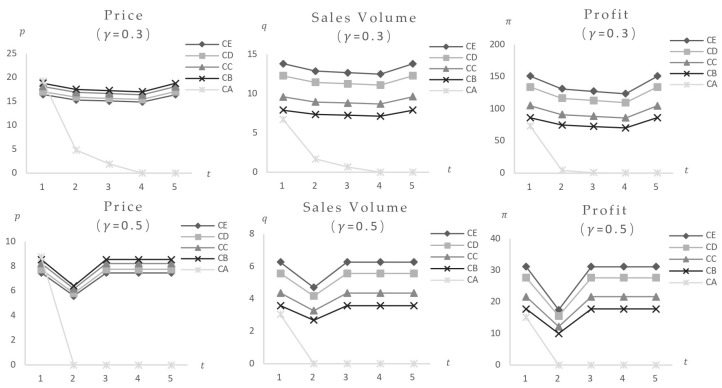
The variation of the equilibrium value of food at the same safety level ($F_{i}$ = 30) when CA safety incident occurs in Food CA ($c_{CA}$ = 2.5) under different levels of consumer safety preferences.

**Figure 9:**
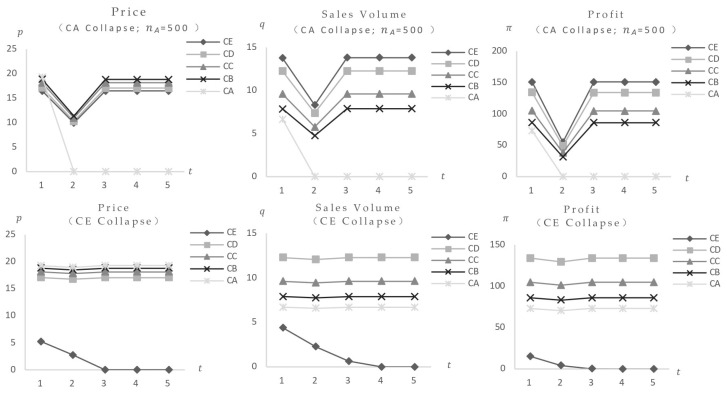
In the event of A safety incident involving Food CE ($c_{CE}$ = 1.5) or A with a higher market share ($c_{CA}$ = 2.5, $n_{CA}$ = 500), the change in the equilibrium value of food at the same safety level ${(F}_{i}$ = 30) (γ = 0.3).

Initial calculations investigate the influence of a single food occurrence. Figure 8 shows the market impact of Food CA, which is distinguished by the highest cost and price. With Food CA ($c_{CA}$ = 2.5) categories set at 40, post-impact equilibrium values for other foods at the same risk level initially declined before increasing. In comparison to pre-impact levels, final stable values showed modest drops in equilibrium price and profit, showing a weak negative effect, while equilibrium sales volume showed a slight increase, demonstrating a weak positive effect. These findings are consistent with higher resident safety preferences and product category values (Figure 9, additional data and figures excluded). The key distinction is the increased market impact and quicker procedure as consumers’ safety preferences and product category values rise.

This analysis shows that in regions with strong resident safety preferences and a high market position for the affected food, market fluctuations have a larger amplitude, shorter impact periods, and a more pronounced rise in sales volume and decline in competitive food prices and profits. Additional calculations for the simultaneous collapse of two food kinds yield consistent results, with precise figures excluded. This may elucidate the reason for the variation in the impact results of the same event across various regions, such as when the concurrent demand for recalled ground beef in the United States was significantly reduced by the *E. coli* recall in most regions of the country. However, the impact was not immediately apparent in certain regions [27]. This study is a valuable addition to the existing research.

Figure 4 and Figure 5 show that when a food safety incident only affects consumers’ risk perception of a certain brand or food type, high-safety foods cause price and profit drops in other same-safety brands while increasing sales volume. Prices, sales volumes, and profitability for foods below this safety limit are all declining. The impact of high-risk foods on same-safety-level foods remains constant, whereas foods at other safety levels show increases in pricing, sales volumes, and profits. This implies that when consumers perceive an increased safety risk in a specific food, they consume more of other brands with the same safety level. Consumers tend to switch to alternative food kinds for high-risk foods, whereas for low-risk foods, they choose the same type of food with higher safety standards.

3. Shock with both consumers’ food safety preferences and food safety varying. Consider the consumers’ food safety preferences γ at values of 0.01, 0.3, 0.5, 0.8, and 0.9. The model calculates the equilibrium value changes in food at each safety level ($F_{i}$ = 30) resulting from one or more food safety incidents.

**Figure 10:**
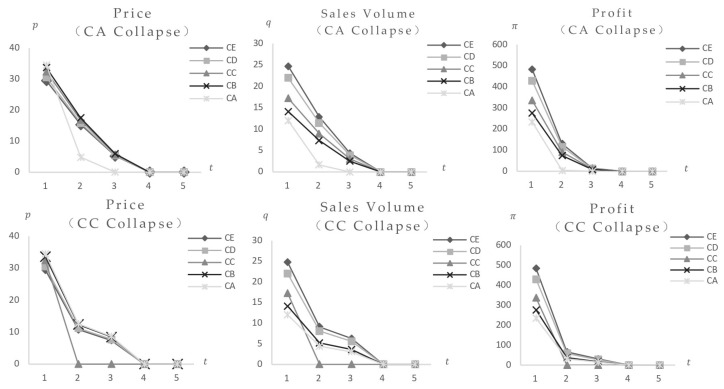
When consumers’ risk perception and preference change simultaneously, safety incidents occur in Food CA ($c_{CA}$ = 2.5) or CC ($c_{CC}$ = 1.5), and the equilibrium value of food at the same safety level changes ($F_{i}$ = 30).

The analysis begins by calculating the outcome of the food explosion and the influence of changes in consumers’ safety preferences. Figure 10 shows the impact of food safety incidents on food safety in Food CA ($c_{CA}$ = 2.5) and Food CC ($c_{CC}$ = 2.5). The findings show that when a food product fails in the market, people’s safety preferences increase, causing other food products with the same risk rating to decrease dramatically until they are eliminated. Once the consequences of food categories CA and CC are compared, it is clear that larger amounts of food collapse correlate with greater market impact. Furthermore, the study of the simultaneous collapse of both food categories validates these conclusions, albeit precise data are not provided here. This analysis shows that food safety incidents affecting specific foods and increasing consumers’ safety preferences have a negative effect on other items in the same safety classification.

These findings explain the detrimental negative effects that food safety incidents have on competing businesses in the same industry, such as the poisoning incident of Chinese imported dumplings that occurred in Japan, where the number of Japanese consumers’ willingness to purchase Chinese food decreased by nearly two-thirds [2].

The findings in Figure 6 show that when a food safety incident increases consumers’ risk perception and safety preferences for specific food types, it causes significant and long-term damage to the entire industry segment, especially when high-safety foods are involved, making industry recovery extremely difficult. This pattern may explain the continued dominance of imported brands in China’s dairy sector following the melamine issue.

## 5. Conclusions and Implications

### 5.1. Research Conclusions

This study presents a general equilibrium model that takes into account consumers’ food safety preferences as well as the variability of food safety risks. The model numerically replicates the spillover effects in the food market that occur as a result of food safety accidents. This study shows that consumers’ risk perceptions and safety preferences play an important role in determining the spread of food safety issues.

The key research findings include the following:

- Once an occurrence merely increases consumers’ safety preferences, all food incurs a negative effect. As safety preferences rise, high-risk foods are eliminated, while high-safety foods settle into a lower equilibrium than before the occurrence.
- Once an occurrence just increases consumers’ risk perceptions of specific meals, the spillover effects become more diverse. Initially, the direction of the impact is contingent upon the safety level of the product in question, as it is not all at the same safety level. Other safety-level foods will be adversely affected if the food in question is classified as a high-safety food. If the food in question is classified as a high-risk food, it will have a beneficial effect on other foods that are classified as safety-level foods. Secondly, non-involved items with the same safety level will have a higher sales volume, but lower pricing and profits. This means that consumers will increase their consumption of other brands or types of food, but because of the drop in prices, firms’ earnings will eventually suffer significant losses.
- Once an occurrence affects both risk perception and safety preferences, it causes negative market effects that are difficult to repair. Only when high-risk food is involved in an occurrence does there exist a recovery possibility.
- The market position of the relevant food (which included two dimensions: market share and safety level) influenced the intensity and duration of the incident’s impact. The larger the market share of the involved food, the greater the incident’s impact and duration. For the same market share, the higher the safety level of the involved food, the greater the impact of the incident.
- The market risk structure can also influence the magnitude and duration of the event shock. The larger the market risk, the greater the event’s impact and duration.

### 5.2. Policy Recommendations

The research conclusions of this article have significant policy implications for the global food market.

Firstly, to avoid food panic and the compound negative effects of risk perception and safety preferences, authorities should promptly notify features contributing to safety concerns in unexpected food occurrences, including specific details of impacted items, enterprises, and industries.

Secondly, increased safety monitoring should concentrate on food products with high energy consumption and pollution levels. Safety issues involving high-safety foods with a large market share might cause severe negative market effects. As a result, regulatory emphasis should focus on industry-leading companies while maintaining strict supervision over organic food certification and inspection systems.

Furthermore, encouraging enterprises to give priority to the production of high-safety food (such as organic food) and developing low-risk food markets is critical. In regions with elevated food market risk structures or lower safety preferences, boosting certified food, traceable food, and other high-safety-level foods advocacy and encouraging enhanced safety preferences can improve market resilience to unforeseen events.

Lastly, even in locations with strong safety preferences and lower market risk structures, close monitoring of high-risk goods is still required to prevent the emergence of chronic safety issues.

### 5.3. Limitations and Prospects

This study simplifies the role of human capital and does not account for variations in the length of the production chain. Future research could explore more complex scenarios in greater depth. With the advancement of social development and the rise in residents’ educational attainment, human capital will play an increasingly important role in enterprise production, potentially influencing both the intensity and the speed of event impacts. Moreover, as the social division of labor deepens, the length of the food supply chain expands. Food safety incidents occurring at different stages of the production chain may exert more complex effects on enterprises [52]. Future research could therefore examine the influence of varying chain lengths and their effects on different stages of the supply chain.

## Figures and Tables

**Table 1:** Basic parameter values of the food market.

Parameters	α	β	ϑ	*X*	*n*
Numerical value	60	0.9	0.5	40	400

**Table 2:** Costs and market structures of different risk foods.

Food		E	D	C	B	A
$F_{i}$		80	60	30	10	0.1
$c_{i}$		0.8	1	1.5	2	2.5
$n_{i}$	High-risk market	200	100	60	30	10
	Low-risk market	10	50	200	100	40

**Table 3:** A group of foods with the same risk ($F_{i}$ = 30) but different costs and their market structures.

Food		CE	CD	CC	CB	CA
$F_{i}$		30	30	30	30	30
$c_{i}$		0.8	1	1.5	2	2.5
$n_{i}$	High-risk market	200	100	60	30	10
	Low-risk market	10	50	200	100	40

## Data Availability

The original contributions presented in this study are included in the article; further inquiries can be directed to the corresponding author.

## Appendix A. Figure 2 Data Source

This data is derived from the annual “Green Food Statistical Annual Report” and “Organic Food Statistical Data” published by the China Green Food Development Center, along with the annual “Statistical Yearbook” published by the National Bureau of Statistics.

## Appendix B. Explanations of the Measurement Units for Each Parameter

Since this paper adopts the numerical simulation method and does not use real data, the units of each variable differ.

γ represents the safety preference of residents, with a value range of [0, 1] and no unit.

The units of price, sales volume, and profit are monetary units, which may be the Renminbi, the US dollar, the British pound, or the currencies of other countries.

Time t is a virtually defined time unit for the convenience of discussing the change process. In reality, it may correspond to days, months, years, etc., and the intervals of time change may not be equal.

The focus of this paper lies in observing the trends and directions of changes after the impact of food safety incidents. Therefore, although the units of each variable in the numerical simulation differ from those in reality, this does not affect the research conclusions of this paper.
